# Dithiocarbamates effectively inhibit the α-carbonic anhydrase from *Neisseria gonorrhoeae*

**DOI:** 10.1080/14756366.2021.1988945

**Published:** 2021-12-11

**Authors:** Simone Giovannuzzi, Nader S. Abutaleb, Chad S. Hewitt, Fabrizio Carta, Alessio Nocentini, Mohamed N. Seleem, Daniel P. Flaherty, Claudiu T. Supuran

**Affiliations:** a Neurofarba Department, Pharmaceutical and Nutraceutical Section, University of Florence, Sesto Fiorentino, Italy; b Department of Biomedical Sciences and Pathobiology, Virginia-Maryland College of Veterinary Medicine, Virginia Polytechnic Institute and State University, Blacksburg, VA, USA; c Department of Medicinal Chemistry and Molecular Pharmacology, College of Pharmacy, Purdue University, West Lafayette, IN, USA; d Center for Emerging Zoonotic and Arthropod-borne Pathogens, Virginia Polytechnic Institute and State University, Blacksburg, VA, USA; e Purdue Institute for Drug Discovery, West Lafayette, IN, USA; f Purdue Institute of Inflammation, Immunology and Infectious Disease, West Lafayette, IN, USA

**Keywords:** Carbonic anhydrase, inhibitor, dithiocarbamate, *Neisseria gonorrhoeae*, antibacterials

## Abstract

Recently, inorganic anions and sulphonamides, two of the main classes of zinc-binding carbonic anhydrase inhibitors (CAIs), were investigated for inhibition of the α-class carbonic anhydrase (CA, EC 4.2.1.1) from *Neisseria gonorrhoeae*, NgCA. As an extension to our previous studies, we report that dithiocarbamates (DTCs) derived from primary or secondary amines constitute a class of efficient inhibitors of NgCA. K_I_s ranging between 83.7 and 827 nM were measured for a series of 31 DTCs that incorporated various aliphatic, aromatic, and heterocyclic scaffolds. A subset of DTCs were selected for antimicrobial testing against *N. gonorrhoeae*, and three molecules displayed minimum inhibitory concentration (MIC) values less than or equal to 8 µg/mL. As NgCA was recently validated as an antibacterial drug target, the DTCs may lead to development of novel antigonococcal agents.

## 1. Introduction

A decase ago, prokaryotic carbonic anhydrases (CAs, EC 4.2.1.1) were proposed as drug targets for development of novel antibacterials^1^. CAs catalyse the interconversion between CO_2_ and bicarbonate, which generate a pH imbalance; CAs are widespread in bacteria and play an important role in various metabolic functions^2^^,^^3^. Bacteria encode at least four genetic families of CAs, including the α-, β-, γ-, and ι-CAs, with many species containing more than one class and more than one CA isoform; however the functions of these different CAs have only recently started to be understood in detail^1–3^. Although comprehensive *in vitro* inhibition studies of bacterial CAs are available^1^^,^^2^, these results have only recenlty been validated *in vivo*. Seminal reports of Flaherty’s and Seleem’s groups showed that in some bacteria, such as in vancomycin-resistant enterococci (VRE) or *Neisseria gonorrhoeae*, clinically used sulphonamide CA inhibitors (CAIs) possess potent antibacterial activity^4^^,^^5^. *N. gonorrhoeae* is a sexually transmitted pathogen that is becoming a global health concern due to increased resistance to a wide range of antibioticsincluding next generation cephalosporins^6^^,^^7^. Acetazolamide, the CAI *par excellence,* and some of its newly designed derivatives were recently shown to be bacteriostatic against *N. gonorrhoeae* with minimum inhibitory concentration values as low as 0.25 μg/mL and no toxicity obseved to host cells^5^. Sulphonamides, of which acetazolamide belongs to, are one of the main classes of CAIs, and their interaction with bacterial CAs from various pathogens has been extensively studied in the last decade^8–11^. As there is an urgent need for novel antibacterials, including antigonococcal agents, a deeper investigation of CA and profiling various classes of CAIs may be of great interest. A previous study of anion inhibitors found interesting inhibitory effects of *N,N*-diethyl-ditiocarbamate [5b], which was as a low micromolar inhibitor of the α-CA *N. gonorrhoeae* (NgCA). Based upon this previous study, we investigated dithiocarbamates as inhibitors of NgCA.

## 2. Materials and methods

### 2.1. Enzymology and CA activity and inhibition measurements

An Applied Photophysics stopped-flow instrument was used to assay the CA- catalysed CO_2_ hydration activity^12^. Phenol red (0.2 mM) was used as a pH indicator, working at the absorbance maximum of 557 nm, with 10 mM HEPES (pH 7.4) as a buffer, and in the presence of 10 mM NaClO_4_ to maintain constant ionic strength, in order to follow the initial rates of the CA-catalysed CO_2_ hydration reaction for a period of 10–100 s. The CO_2_ concentrations ranged from 1.7 to 17 mM for the determination of the kinetic parameters and inhibition constants. For each inhibitor, at least six traces of the initial 5–10% of the reaction were used to determine the initial velocity. The uncatalyzed rates were determined in the same manner and subtracted from the total observed rates. Stock solutions of inhibitors (10–20 mM) were prepared in distilled-deionized water, and dilutions up to 0.01 µM were done thereafter with the assay buffer. Inhibitor and enzyme solutions were preincubated together for 15 min at room temperature prior to the assay, in order to allow for the formation of the E-I complex. The inhibition constants were obtained by non-linear least-squares methods using Prism 3 and the Cheng-Prusoff equation, as reported earlier^13^^,^^14^, and represent the mean from at least three different determinations. The NgCA concentration in the assay system was 6.3 nM. The NgCA used was a recombinant enzyme obtained in-house, as described earlier^5^^,^^15^^,^^16^.

### 2.2. Chemistry

DTCs **1–30** were previosuly reported by one of our groups^17^^,^^18^ and were of > 99% purity. DTC **31**, acetazolamide, buffers and other reagents are commercially available from Sigma-Aldrich (Milan, Italy).

### 2.3. Bacterial strains and media

Strains and media used in this study were previously reported by our group^5^^,^^19^. *N. gonorrhoeae* strains used in the study were clinical isolates obtained from the Centres for Disease Control and Prevention (CDC). Media and reagents were purchased commercially: brucella broth, IsoVitaleX, and chocolate II agar plates (Becton, Dickinson and Company, Cockeysville, MD, USA), yeast extract and dextrose (Fisher Bioreagents, Fairlawn, NJ, USA), protease peptone (Oxoid, Lenexa, KS, USA), haematin, pyridoxal, and nicotinamide adenine dinucleotide (NAD) (Chem-Impex International, Wood Dale, IL, USA), and phosphate buffered saline (PBS) (Corning, Manassas, VA, USA).

### 2.4. *Antibacterial activity of DTCs against* N. gonorrhoeae *strains*

The (MICs of DTCs compounds were carried out using the broth microdilution method as described previously^5^^,^^19^. Briefly, bacterial strains were grown for 24 h on GC chocolate agar II, at 37° C in presence of 5% CO_2_. Then a bacterial suspension equivalent to 1.0 McFarland standard was prepared and diluted in brucella broth supplemented with yeast extract, protease peptone, haematin, pyridoxal, NAD, and IsoVitaleX, to achieve a bacterial concentration of about 1 × 10^6^ CFU/mL. Test agents were added in the 96-well plates and serially diluted along the plates. Plates were then, incubated for 24 h at 37° C either aerobically or in the presence of 5% CO_2_ before determining the MICs as observed visually.

## 3. Results and discussion

**Figure 1.**
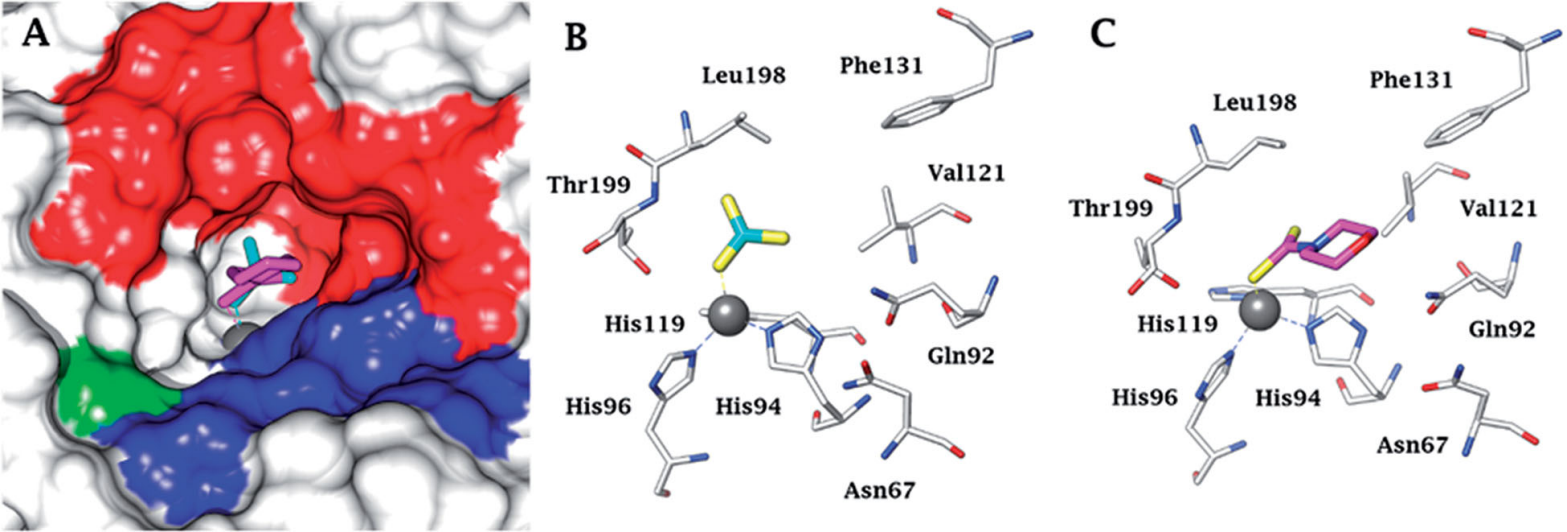
(A) Surface representation of hCA II active site in adduct with superimposed trithiocarbonate (cyan, PDB 3K7K) and the DTC morpholinocarbodithioate **23** (magenta, PDB 3P5A). The hydrophobic half of the CA active site is shown in red, and the hydrophilic one in blue; the proton shuttle residue His64 is shown in green. Cartoon view of hCA II active site in complex with B) trithiocarbonate and C) DTC **23**.

Sulphonamide-type CAIs were first used to inhibit growth of *N. gonorrhoeae in vitro* in the 1960s; however, it was not untill the 1990s that Carter’s group reported the presumed presence of CAs in *N. gonorrhoeae* by using a monospecific antibody prepared against the purified *Neisseria sicca* enzyme^15^. This enzyme was thereafter purified and characterised in 1997 by Lindskog’s group^16^, who showed that NgCA is an α-class enzyme that possesses a high catalytic activity, with a k_cat_ for the CO_2_ hydration reaction of 1.7 × 10^6^ s^−1^ ^17^. The same group showed that NgCA was inhibited by metal complexing anions such as cyanide, cyanate, thiocyanate, and azide (as determined by using the esterase actvity of the enzyme with 4-nitrophenyl acetate as a substrate^16^) as well as by the sulphonamide acetazolamide (5-acetamido-1,3,4-thiadiazole-2-sulphonamide)^16^. Thereafter, we reported a comprehensive anion inhibition study of NgCA [5b], which found that the most effective inhibitors were sulfamide, sulphamic acid, and *N,N*-diethyl-dithiocarbamate. This compound possesses the CS_2_^−^ zinc-binding group (ZBG), also present in trithiocarbonate (TTC)^17^, which has been shown via X-ray crystallography on human CAs (hCAs) to bind in a monodentate fashion to the metal ion from the enzyme’s active site to displace the nucleophile (water or hydroxide ion) that is essential in the catalytic process^17^. The X-ray structure of TTC bound to hCA II led thereafter to the discovery of DTCs and their derivatives (monothiocarbamates and xanthates) as potent CAIs^18^^,^^20^. X-ray crystallography of some DTCs bound to hCA II demonstrated that their ZBG is coordinated in a monodentate fashion to the metal ion whereas the organic scaffold participates in a range of favourable interactions with the active site amino acid residues^18^ – Figure 1.

Thus, we decided to investigate a series of previously reported DTCs^18^, types **1–30** together with the *N,N*-diethyl derivative **31**, for their interaction with NgCA (Table 1). The following structure-activity relationship (SAR) may be observed from the data presented in Table 1:

**Table 1.** Inhibition constants (K_I_s) of DTC inhibitors **1–31** against hCA I, II, and NgCA by a stopped flow CO_2_ hydration assay, using acetazolamide (AAZ) as the standard drug^12^.

DTC	Structure	K_i_ (nM)^a^		
		hCA I	hCA II	NgCA
**1**	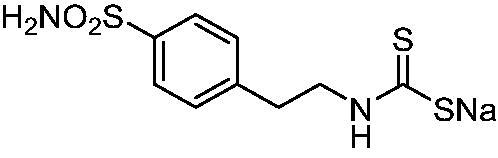	97.5	48.1	83.7
**2**	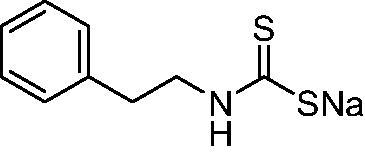	425	107.0	259
**3**	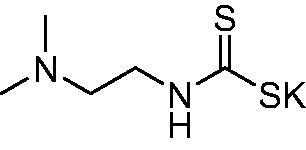	85.9	35.8	568
**4**	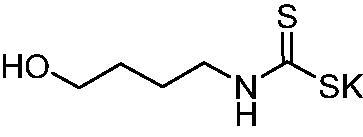	295	24.3	438
**5**	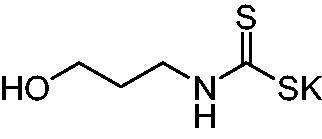	706	41.7	413
**6**	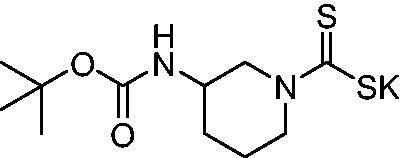	683	13.2	538
**7**	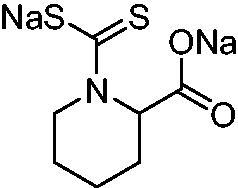	485	80.1	827
**8**	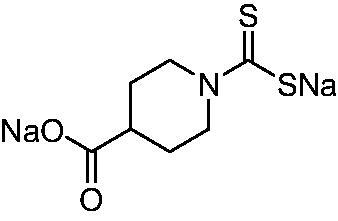	337	78.7	514
**9**	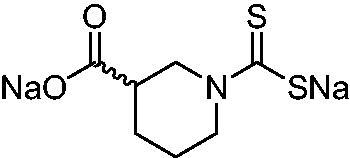	290	45.4	297
**10**	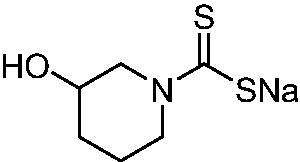	428	60.7	367
**11**	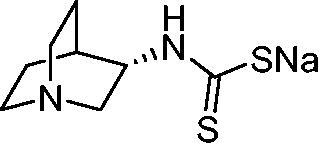	615	65.9	473
**12**	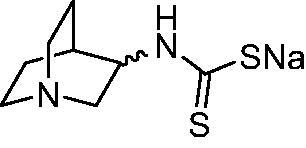	494	48.7	482
**13**	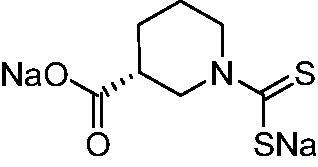	496	80.5	242
**14**	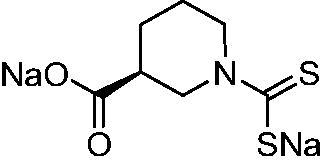	109	8.9	335
**15**	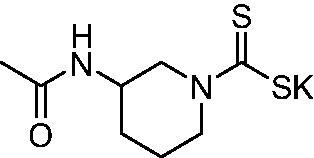	910	47.9	451
**16**	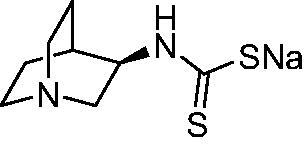	240	18.9	518
**17**	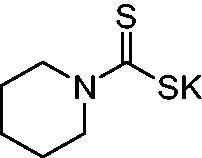	252	30.1	731
**18**	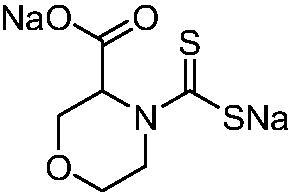	84.7	78.5	672
**19**	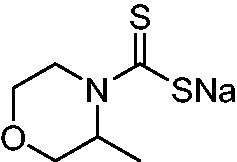	434	60.2	723
**20**	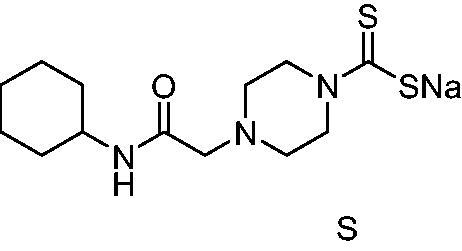	415	67.2	84.4
**21**	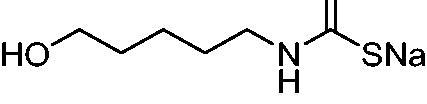	66.5	17.3	454
**22**	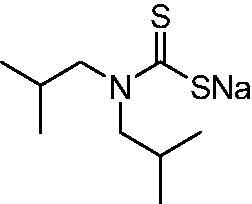	0.97	0.95	554
**23**	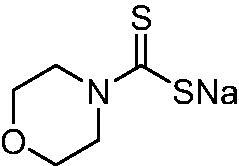	0.88	0.95	483
**24**	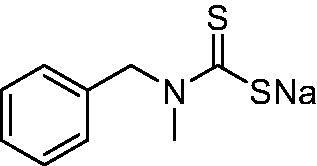	69.9	25.4	654
**25**	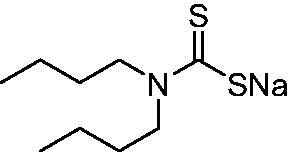	43.1	50.9	460
**26**	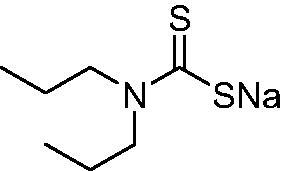	1838	55.5	522
**27**	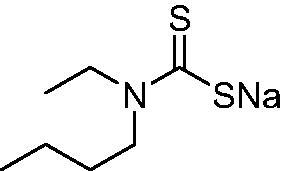	157	27.8	577
**28**	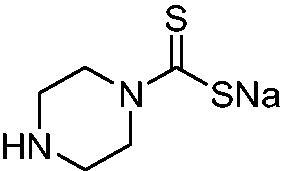	31.9	13.5	276
**29**	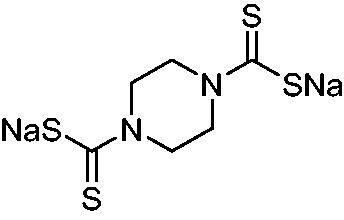	12.6	0.92	136
**30**	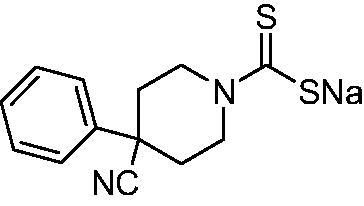	48.4	40.8	365
**31^b^**	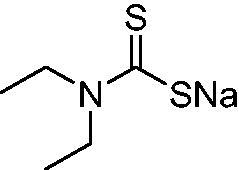	790	3100	5100
**AAZ**	–	250	12.0	75.0

^a^Mean from three different assays, determined using a stopped flow technique (errors were in the range of ± 5–10% of the reported values); ^b^from ref. [5b].

- The most effective NgCA inhibitors among the investigated DTCs were compounds **1, 20** and **29**, which showed K_I_s in the range of 83.7–136 nM. It is interesting to note that both **20** and **29** possess the same scaffold of piperazine-dithiocarbamate. However, in the case of **29** a second DTC function is incorporated, whereas for **20,** a bulkier cyclohexyl-aminocarbonylmethyl moiety is present. This leads to an increased inhibitory effect in the case of **20** compared to **29** (84.4 versus 136 nM, Table 1), probably due to favourable contacts between the bulky tail and amino acid residues from the active site. The second observation pertains to compounds **1** and **2**. Derivative **1** incorporated two ZBGs, the DTC and the sulphonamide ones, whereas the second structurally related derivative (**2**) lacks the sulphonamide moiety. It is likely in the case of **1** that sulphonamide is the dominant interacting group and participates in the enzyme inhibition process by binding to the zinc ion in the active site. This is however impossible for **2**, which exhibited 3.1 times weaker NgCA inhibitory activity compared to **1**. However, derivative **2** still significantly inhibited the NgCA CO_2_ hydrase activity with a K_I_ of 259 nM.
- Another small group of DTCs, including **2, 9, 13,** and **28** showed K_I_s in the range of 242 – 297 nM, which indicates that they are effective NgCA inhibitors. The next most effective inhibitors showed K_I_s between 300 and 500 nM and included **4, 5, 10–12, 14, 15, 21, 23, 25,** and **30**. These compounds incorporated a variety of diverse aliphatic, aromatic, and heterocyclic scaffolds, and are derivatives of both primary and secondary amines. This proves that many diverse chemical entities may lead to the development of efficient DTC inhibitors of NgCA (Table 1).
- The least effective inhibitors were **3**, **6–8**, **16–19**, **22**, **26**, and **27**, which showed K_I_s in the range of 514–827 nM. Finally, **31**, the lead compound was the least effective DTC inhibitor, with a K_I_ of 5100 nM. In contrast, acetazolamide, a sulphonamide derivative, was an effective NgCA inhibitor, with an activity in the same range as the most effective DTCs mentioned above (Table 1).
- Many of the investigated DTCs were much more effective as inhibitors against hCA II than NgCA, whereas their activity on hCA I was in the same range as against the bacterial enzyme, i.e. in the high nanomolar range.

A subset of DTCs were selected for antibacterial testing against three clinical strains of *N. gonorrhoeae*. It has previously been established that bacteria will become less susceptible to CAIs in conditions that contain elevated levels of CO_2_^21^_._ Molecules were assayed in both ambient air conditions as well as conditions containing 5% CO_2_ to assess for activity at the proposed intracellular NgCA. The three strains tested displayed reduced susceptibility towards the molecules under elevated CO_2_ conditions suggesting that inhibition of NgCA is, at least partially, responsible for the antimicrobial activity of these molecules. The control antibiotic azithromycin, which has a different mechanism of action, did not display differential activity based on the culture conditions. This result provides confidence that the difference in CO_2_ levels did not have unintended effects on the bacteria that would result in non-specific reduced susceptibility to the test agent.

It was observed that in this cohort, three DTCs, **1**, **22**, and **24** exhibited moderate antigonococcal activity. DTC **1** was the most potent molecule with a MIC value of 1–2 µg/mL against *N. gonorrhoeae* (Table 2). This was followed by **22** (MIC = 2–4 µg/mL) and **24** (MIC =4–8 µg/mL). DTCs **23** and **25** each displayed weak antibacterial activity against *N. gonorrhoeae* with MIC values ranging from 8 to 32 µg/mL. It is interesting to note that while **1** was the most potent molecule against both NgCA and *N. gonorrhoeae,* the DTCs that exhibited moderate potency against *N. gonorrhoeae* (**22** and **23**) were among the weaker analogues versus NgCA (K_I_s > 500 nM). Moreover, the weakest DTCs, in terms of antigonococcal activity, were **23**, **25**, **28**, **29**, and **30** with MIC values > 8 µg/mL; however, these molecules were more potent inhibitor of NgCA with activities in the range of 136 − 460 nM. Several of these molecules contain polar functional groups such as morpholine (**23**), piperazine (**28**) and Di-DTC (**29**) moieties that may have an adverse effect on molecule accumulation within the Gram-negative bacterial cell, thus leading to reduced antigonococcal activity. As for DTC **25**, this molecule contains hydrophobic linear alkyl chains that give rise to additional rotatable bonds that also may have an adverse effect on accumulation into Gram-negative bacterial cells^22^^,^^23^. In summary, while the DTCs displayed moderate-to-weak antibacterial activity against the *N. gonorrhoeae* strains tested, the data does suggest that the DTC functionality may be a useful modification to incorporate into a drug design campaign for development of new anti-gonococcal agents.

**Table 2.** Minimum inhibitory concentrations of DTCs versus *N. gonorrhoeae* clinical isolates.

Test agents/ Control antibiotics	MIC values versus *Neisseria gonorrhoeae* strains (µg/mL)					
	CDC 178		CDC 181		CDC 194	
	5% CO_2_^a^	Ambient air^b^	5% CO_2_^a^	Ambient air^b^	5% CO_2_^a^	Ambient air^b^
**1**	16	2	16	1	16	2
**3**	>64	>64	>64	>64	>64	64
**22**	>64	4	>64	2	>64	4
**23**	>64	32	>64	16	>64	8
**24**	32	8	32	4	32	8
**25**	>64	32	>64	16	>64	16
**28**	>64	>64	>64	>64	>64	64
**29**	>64	>64	>64	>64	>64	64
**30**	>64	64	>64	64	>64	64
AAZ	>64	2	>64	4	>64	2
Azithromycin	2	2	>64	>64	1	0.5

^a^Indicates incubation in presence of 5% CO_2_. ^b^Indicates in ambient air.

## 4. Conclusions

NgCA, a high-activity α-CA present in the genome of *N. gonorrhoeae*, was investigated for potential inhibition by a series of 31 DTCs derived from both primary and secondary amines. NgCA was inhibited by all investigated derivatives, with K_I_s in the range of 83.7 nM − 5.1 µM. The most effective NgCA inhibitors were contained piperazine-dithiocarbamates that showed activity with K_I_s < 140 nM; however, these molecules did not display antibacterial activity *in vitro* against *N. gonorrhoeae*. Conversely, DTCs containing more hydrophobic amines did exhibit moderate antibacterial activity even though these analogs possessed reduced NgCA activity. This data suggests that DTCs could be incorporated as the zinc-binding groups in place of sulphonamides, into more traditional CAI molecular scaffolds. Since antibiotic resistance is well documented against many *N. gonorrhoeae* strains worldwide, finding alternative chemotypes to presently used drugs is relevant. Our study provides interesting steps regarding developing these types of enzyme inhibitors.
